# Research on schedling optimization of four-way shuttle-based storage and retrieval systems

**DOI:** 10.1038/s41598-023-31050-8

**Published:** 2023-03-10

**Authors:** Jia Mao, Jinyuan Cheng, Xiangyu Li, Baogui Cao

**Affiliations:** 1 School of Transportation, Jilin University, Changchun, 130022 China; 2 College of Automotive Engineering, Jilin University, Changchun, China

**Keywords:** Energy science and technology, Engineering, Mathematics and computing

## Abstract

In this paper, we take the four-way shuttle system as the research object and establish the mathematical model of scheduling optimization based on the minimum time for the in/out operation optimization and path optimization scheduling problems of the four-way shuttle system. An improved genetic algorithm is used to solve the task planning, and an improved A* algorithm is used to solve the path optimization within the shelf level. The conflicts generated by the parallel operation of the four-way shuttle system are classified, and the improved A* algorithm based on the time window method is constructed for path optimization through the dynamic graph theory method to seek safe conflict-free paths. Through simulation example analysis, it is verified that the improved A* algorithm proposed in this paper has obvious optimization effect on the model of this paper.

Warehouse logistics has entered the era of automated system integration, with high-rise three-dimensional shelves as the main storage equipment has developed into the main way of intelligent logistics system storage. The main body of work also has shelf storage turned into robots or shuttle + shelves. The storage system with integrated hardware and software such as shelf + shuttle + hoist + picking system + warehouse management system has become one of the main storage modes. In order to improve the demand for urban last-mile delivery time and efficiency, a new type of shuttle system—four-way shuttle system is increasingly popular in use.

As an upgrade of the shuttle system, the four-way shuttle system has characteristics, such as longitudinal and transverse lanes between shelves and cross nodes between lanes. Not only can the trolley run in four directions in a single layer of shelves but it can also run with the hoist to complete a layer change, which is important for improving the stability of storage system operation, work efficiency, and reducing operation production costs. However, with the expansion of inbound and outbound tasks and the increase in the number of available four-way shuttles, the complexity of task allocation and scheduling multivehicle parallel operations of four-way shuttles has increased. Planning a collision-free and conflict-free shortest optimal path for multiple four-way shuttles in a fixed warehouse scenario is a difficult problem in the study of four-way shuttle systems. The four-way shuttle system studied in this paper is a single-depth racking configuration with a row of high-rise racks on each side of each aisle and a hoist at the end of the aisle to match the four-way shuttle's layer change operation. Single-depth racking is suitable for storing small quantities and multiple varieties of small items, and this system requires more main tracks, which crowds the storage space and reduces the storage space utilisation. However, this storage layout stores small-lot, multivariety goods in a flexible access mode, and multiple four-way shuttles can be used to operate in parallel, improving the efficiency of access operations. The floor plan of the four-way shuttle system studied in this paper is shown in Fig. 1.

**Figure 1:**
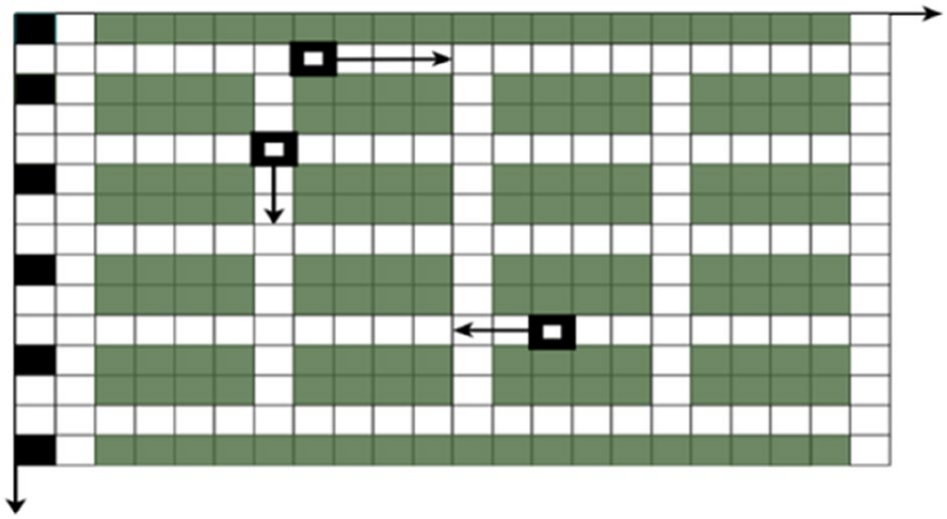
Plan layout of the four-way shuttle system studied in this paper.

This paper provides task scheduling and optimal management of a four-way shuttle storage system to reduce operating time and improve the warehouse’s operational efficiency by making rational use of the coordination between existing equipment without adding investment into the equipment. Automatic access equipment in the literature mainly consists of stacker cranes and shuttles or a combination of both, but less research has been done on four-way shuttle systems. The scheduling problem is more complex because the four-way shuttle can run in longitudinal, lateral and vertical three-dimensional directions to reach any position of the goods^1–6^.

## Related works

The most common types of automated stereo warehouses are AS/RS, SBS/RS, AVS/RS. AS/RS systems are relatively inexpensive to implement in real industrial production, but such systems are less flexible and AS/RS can only feed single- or double-depth racks. As a result, the number of aisles increases with the size of the warehouse, resulting in poor space utilization. In the scientific literature, research on AVS/RS can be broadly divided into interchangeable and non-interchangeable systems. The former is that the trolley can move up and down to different layers with the hoist. The latter is a system in which the trolley is assigned to a specific layer of AVS/RS for operation and cannot move with the hoist.SBS/RS system is an automatic storage system with shuttle car as the core. This system is a new technology in AVS/RS and requires a shuttle car on each layer. Due to its high initial investment cost, it is mainly suitable for small load warehouses. Scholars have mostly focused on AVS/RS and SBS/RS systems for storage systems, and less on four-way shuttle scheduling optimization^7–10^. In^11^ the reference, shuttle scheduling theory was classified according to different scheduling situations and focused on multiple shuttle selections in cases of single task requests versus single shuttle selections in cases of multiple task requests. A fuzzy classification algorithm was applied to determine the probability of job task occurrence, and then the existing job tasks and the job tasks that needed to be generated were considered to solve the dynamic multivehicle path optimisation problem using a genetic algorithm to assign shuttle delivery more rationally^12^. In^13^ the reference, the genetic algorithm and random storage strategy were used to develop a double-loop mode to order the tasks for the AS/RS, and the genetic algorithm model was proposed to simultaneously determine the positions and order of tasks. The model was superior to two greedy heuristic algorithms. The task scheduling problem was transformed between shuttles and lifts into an assembly line parallel operation problem based on the motion characteristics of shuttles and lifts. A scheduling task queue model was generated within a specified time window^14^. Many scholars have considered the application of genetic algorithms for solving models in vehicle scheduling problems but have rarely considered the improvement of solving shuttle bus scheduling models using genetic operators and fitness functions.

Shuttle scheduling optimisation is the focus and difficulty of shuttle system research. Among them, path optimisation is another important method of improving the efficiency of the system. The core of path optimisation is the algorithm design. Traditional algorithms mainly include forbidden search algorithms and simulated annealing methods^15–19^. The simulated annealing algorithm is a local optimum method, which makes it is easy to jump out of the local optimum solution and slow to converge, and it has some influence on the solution accuracy^20–24^. In the reference^25^, an adaptive variable neighbourhood search algorithm and Lagrangian relaxation algorithm were developed to address large instances by establishing a biobjective integer programming model of time and energy. By studying the shared storage system, the crane scheduling problem, as a special type of fault-tolerant vehicle routing problem, was reformulated so that it could be used to solve some open problems regarding the time complexity of the associated geometric routing problem^26^. In the reference^27^, automated guided vehicles, lifts and shuttles were studied for an integrated optimisation problem. A mixed-integer programming model was proposed to optimise pallet assignment to related equipment and storage locations during the inbound process, and an algorithm based on variable neighbourhood search was developed to solve the model efficiently. The reference^28^ considerded a multi-aisle AS/RS with a multishift S/R machine and performed system optimisation using a genetic algorithm. To solve the conflict deadlock problem caused by the simultaneous operation of multiple shuttles, Li and Roy used a zone control method, dividing the storage system into nonoverlapping zones and allowing only one four-way shuttle to perform the task in each zone^29,30^.

Scholars have researched shuttle systems quite maturely but focused more on scheduling shuttles, favouring the normal scheduling operation of the system under multiple objectives and rarely involving system deadlocks, traffic control, etc. In terms of scheduling optimisation, studies have mainly focused on single shuttle systems and storage racking with one shuttle for access tasks in each area, with scheduling models limited to single-level operations or to one lift serving several fixed aisles. To solve the conflict deadlock problem, the area control method and the predictive control method can increase the intermediate link of the operation task and limit the scope of shuttle operation, thus reducing the operational efficiency of the storage system. The four-way shuttle systems can dynamically adjust the operation task according to the load demand, which has strong flexibility, but the parallel operation of multiple shuttles on the same level may generate path conflicts, and the system control scheduling is more complicated. Therefore, the research problem of this paper is to take the four-way shuttle system as the research object, and establish a mathematical model based on the shortest time optimization for scheduling based on the optimization of access operations and path optimization scheduling for multiple four-way shuttles and multiple lifts in the four-way shuttle system. Combining the characteristics of the model, an improved genetic algorithm is used to solve the task planning and improved A* algorithm for path optimization within the shelf level.

### Main contributions

- The innovative algorithm design of a four-way shuttle car and hoist operating in parallel and four-way shuttle car automatic path finding without motion direction restriction is proposed.
- The mathematical function is established based on the shortest time objective optimisation. When combined with the genetic algorithm, it is easy to find the best multipoint search characteristics with the use of the improved genetic algorithm of local path planning to find each shuttle operation path and through the Python software simulation.
- The A* algorithm path finding is improved and enhanced to consider congestion avoidance on the basis of the shortest path. The improved A* algorithm based on dynamic graph theory and time windows is proposed to solve the collision locking problem when multiple shuttle vehicles work together in the same layer for the path conflict problem of multiple four-way shuttle vehicles. The time-consuming shortest path trajectory of four-way shuttle vehicles in the same layer is obtained.
- To provide theoretical support for the four-way shuttle storage system, we provide a theoretical basis for storage planning, design layout and task scheduling, which can improve the space utilisation, task rationality and path optimisation of the four-way shuttle system.

The rest of this paper is organised as follows. The “Building the model” section provides the assumptions of the proposed model and the formulation of the model design applied in this study. The framework of the algorithm and the specific formulation of the improved algorithm are given in the “Proposed algorithm” section. A specific application example of the algorithm proposed in the paper is presented in the “Application example analysis” section. The “Conclusion” section concludes the paper.

## Building the model

### Model assumptions

To better study the four-way shuttle system scheduling optimisation model better, the following assumptions are made for the four-way shuttle system in this paper:

1. The shelves and compartments have the same specifications, each aisle has the same width, and the goods are stored in a random storage strategy.
2. Inbound and outbound operation tasks request that four-way shuttles and hoists work together subject to the FCFS strategy.
3. Each row of shelves has $M$ levels and $N$ columns with fixed length, width and height.
4. Only one four-way shuttle is allowed to operate in the same section of the aisle at the same time.
5. The hoist and the four-way shuttle are in uniformly accelerated motion.
6. The four-way shuttle can only carry a single piece of cargo at a time and the running process is not interrupted. Each hoist can only load one four-way shuttle or a single piece of cargo at a time, and the initial position of the four-way shuttle is the final target cargo position of the last task. The initial position of the hoist is the final target cargo level of the last task at the end of the aisle hoist entrance.
7. The amount of time that the shuttle takes to access goods and the amount of time it takes to enter and leave the hoist are neglected, the shuttle turning time is the same as the constant value.

## Basic parameters

**Table:** 

Parameters	Meaning
${R}_{count}$	Total number of 4-way shuttles of the system ($count=\mathrm{1,2},3\dots n$)
${E}_{count}$	Total number of system hoists ($count=\mathrm{1,2},3\dots m$)
*H*	The height of each shelf
${J}_{count}$	The total number of operational tasks ($count=\mathrm{1,2},3\dots k$)
${W}_{T}$	The width of individual aisles
*M*	The number of shelf levels
*L*	The length of individual cargo compartments
${W}_{C}$	The width of individual cargo compartments
${V}_{Rm}$	the maximum speed of the four-way shuttle
${a}_{R}$	The acceleration of the four-way shuttle
${V}_{Em}$	Maximum maximum speed of the hoist
${a}_{E}$	Acceleration of the hoist
${T}_{2},{T}_{3}$	time function expression is similar in meaning to ${T}_{1}$
${E}_{avaliable}$	Number of available hoists in the system
${R}_{avaliable}$	Number of four-way shuttles available in the system
${M}_{J}$	The target level of the inbound task J is on $(M=\mathrm{1,2},\dots ,M)$
${M}_{R}$	Four-way shuttle car R last operation completed in the floor $(M=\mathrm{1,2},\dots ,M)$
${T}_{1Ei}$	Time taken for hoist i to run unloaded to the warehouse floor inlet $\left(i\subseteq \mathrm{1,2},\dots {E}_{count}\right)$
${T}_{2Ei}$	Time taken to run the lift i load to the level where the target level is located $\left(i\subseteq \mathrm{1,2},\dots {E}_{count}\right)$
${T}_{1RJ}$	Four-way shuttle j time spent running unloaded to the hoist opening at the end of the tunnel $\left(j\subseteq \mathrm{1,2},\dots {R}_{count}\right)$;
${T}_{2RJ}$	Four-way shuttle j Time taken to deliver the load to the target bay $\left(j\subseteq \mathrm{1,2},\dots {R}_{count}\right)$
${T}_{3Ei}$	Time taken to run the hoist i unloaded to the level where the 4-way shuttle is located $\left(i\subseteq \mathrm{1,2},\dots {E}_{count}\right)$;
${T}_{4Ei}$	Time to transport the lift i load four-way shuttle to the floor where the target cargo level is located
${T}_{1Ek}$	Elevator k time spent running empty to the warehouse floor inlet $\left(k\subseteq \mathrm{1,2},\dots {E}_{count}\right)$
${T}_{2Ek}$	Elevator k Time taken to run the load to the level where the target level is located $\left(k\subseteq \mathrm{1,2},\dots {E}_{count}\right)$
${T}_{1Ei}^{\mathrm{^{\prime}}}$	Time from hoist $i$ empty to the floor where the outgoing cargo level is located $\left(i\subseteq \mathrm{1,2},\dots {E}_{count}\right)$
${T}_{1Rj}^{\mathrm{^{\prime}}}$	Time taken for the four-way shuttle $j$ to reach the target cargo space empty $\left(j\subseteq \mathrm{1,2},\dots {R}_{count}\right)$
${T}_{2Rj}^{\mathrm{^{\prime}}}$	The time taken by the four-way shuttle $j$ to pick up and deliver the goods to the hoist $\left(j\subseteq \mathrm{1,2},\dots {R}_{count}\right)$
${T}_{2Ei}^{\mathrm{^{\prime}}}$	Time taken by hoist $i$ to transport goods to export $\left(i\subseteq \mathrm{1,2},\dots {E}_{count}\right)$
${T}_{3Ei}^{\mathrm{^{\prime}}}$	Time spent with hoist $i$ empty to other available trolley levels $\left(i\subseteq \mathrm{1,2},\dots {E}_{count}\right)$
${T}_{3Rj}^{\mathrm{^{\prime}}}$	Time taken for the four-way shuttle $j$ to reach the hoist opening empty $\left(j\subseteq \mathrm{1,2},\dots {R}_{count}\right)$
${T}_{1Ei}^{\mathrm{^{\prime}}}$	Time taken for hoist $j$ to load a four-way shuttle to the level where the target level is located $\left(i\subseteq \mathrm{1,2},\dots {E}_{count}\right)$
${T}_{S}^{\mathrm{^{\prime}}}$	The start time of the first outbound operation
${T}_{E}^{\mathrm{^{\prime}}}$	The time to complete the last outbound operation
${f}_{max}$	Maximum fitness value in the population
$f$	The adaptation value of the larger of the two individuals to cross in the population
${f}_{agv}$	Average fitness value of all individuals in the population
${f}^{\mathrm{^{\prime}}}$	The fitness value of the individuals in the population to be mutated
$N$	Number of single-row cargo compartments
$K$	Number of lanes

**Figure 2:**
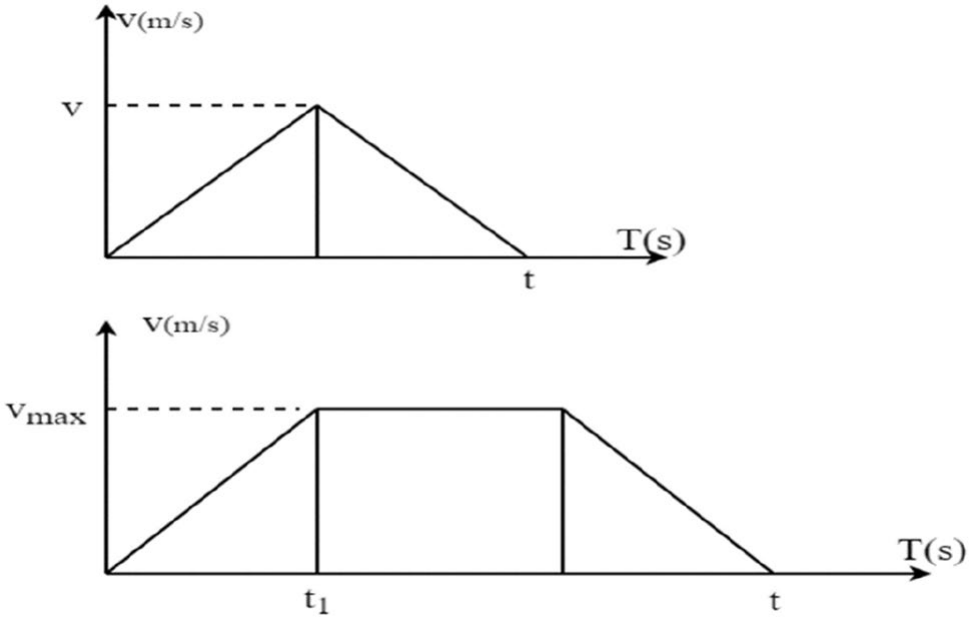
Four-way shuttle running speed versus time.

In the four-way shuttle system studied in this paper, the speed and time relationship of the shuttle operation is shown in Fig. 2. Based on the assumptions, the aisle length, cross-aisle length and shelf height required for the four-way shuttle and hoist to accelerate to the maximum speed and then decelerate to 0 are $S_{Rm} = \left| {\frac{{V_{Rm}^{2} }}{{a_{R} }}} \right|, \;{ }S_{Cm} = \left| {\frac{{V_{Rm}^{2} }}{{a_{R} }}} \right|{,}\;{ }H_{Em} = \left| {\frac{{V_{Rm}^{2} }}{{a_{E} }}} \right|$. The length of each compartment, the width of each compartment and the width of the aisle and the height of each shelf are known, so the minimum number of compartments and the number of levels needed are $L_{Rm} = \left| {\frac{{S_{Rm} }}{L}} \right|,\;{ }L_{Cm} = \left| {\frac{{S_{Rm} }}{{2W_{C} + W_{T} }}} \right|,\;L_{Em} = \frac{{H_{Rm} }}{H}$^31^.

- Time of horizontal direction
  1. If the initial position of the four-way shuttle to the target cargo position or the end of the tunnel hoist opens in the same tunnel. That is, the time required to run the shuttle from column $x$ to column ${x}^{\mathrm{^{\prime}}}$ or from column $x$ to column 0 is:
    1 $$t_{{xx^{\prime}}} = \left\{ {\begin{array}{*{20}l} {\sqrt {\frac{{4\left| {x - x^{\prime}} \right|L}}{{a_{R} }},} } \hfill & {\left| {x - x^{\prime}} \right| \le L_{{Rm}} } \hfill \\ {\frac{{\left| {x - x^{\prime}} \right|L}}{{V_{{Rm}} }} + \frac{{V_{{Rm}} }}{{a_{R} }},} \hfill & {L_{{Rm}} < \left| {x - x^{\prime}} \right| \le N} \hfill \\ \end{array} } \right.$$

Or
    2 $${\text{t}}_{{{\text{x}}0}} = \left\{ {\begin{array}{*{20}l} {\sqrt {\frac{{4{\text{xL}}}}{{{\text{a}}_{{\text{R}}} }}} } \hfill & {x \le {\text{L}}_{{{\text{Rm}}}} } \hfill \\ {\frac{{{\text{xL}}}}{{{\text{V}}_{{{\text{Rm}}}} }} + \frac{{{\text{V}}_{{{\text{Rm}}}} }}{{{\text{a}}_{{\text{R}}} }}} \hfill & {{\text{L}}_{{{\text{Rm}}}} x \le N} \hfill \\ \end{array} } \right.$$
  (B) When the initial position of the four-way shuttle and the target cargo position or the position of the four-way shuttle and the end-of-aisle hoist are not in the same aisle, that is from column $x$ to column ${x}^{\mathrm{^{\prime}}}$ by many segments of straight travel time, and from aisle $y$ to aisle ${y}^{\mathrm{^{\prime}}}$ by several segments of switching aisle time, so the time of shuttle operation is the sum of a number of segments of straight travel time and a number of segments of switching aisle time. The time for each straight section is similar to the solution in (1), and the time for each section to switch lanes is:
    3 $$t_{{yy^{\prime}}} = \left\{ {\begin{array}{*{20}l} {\sqrt {\frac{{4\left| {y - y^{\prime}} \right|\left( {2W_{C} + W_{T} } \right)}}{{a_{R} }}} ,} \hfill & {\left| {y - y^{\prime}} \right| \le L_{{Cm}} } \hfill \\ {\frac{{\left| {y - y^{\prime}} \right|\left( {2W_{C} + W_{T} } \right)}}{{V_{{Rm}} }} + \frac{{V_{{Rm}} }}{{a_{R} }},} \hfill & {L_{{Cm}} < \left| {y - y^{\prime}} \right| \le K} \hfill \\ \end{array} } \right.$$

If the aisle goes from the first aisle to the other $y$ aisle, the time required for the four-way shuttle to run is:

4 $$t_{{y0}} = \left\{ {\begin{array}{*{20}l} {\sqrt {\frac{{4\left| y \right|\left( {2W_{C} + W_{T} } \right)}}{{a_{R} }}} } \hfill & {\left| y \right| \le L_{{Cm}} } \hfill \\ {\frac{{\left| y \right|\left( {2W_{C} + W_{T} } \right)}}{{V_{{Rm}} }} + \frac{{V_{{Rm}} }}{{a_{R} }}} \hfill & {L_{{Cm}} < \left| y \right| \le K} \hfill \\ \end{array} } \right.$$

b. Time in the vertical direction of the hoist
  The initial position of the end-of-aisle hoist to the first floor of the warehouse pick up, that is, from the $m$ floor to the first floor or from the first floor to ${m}^{\mathrm{^{\prime}}}$ time is:
  5 $$t_{{m0}} = \left\{ {\begin{array}{*{20}l} {\sqrt {\frac{{4mH}}{{a_{R} }}} } \hfill & {m \le L_{{Em}} } \hfill \\ {\frac{{mH}}{{V_{{Em}} }} + \frac{{V_{{Em}} }}{{a_{E} }}} \hfill & {L_{{Em}} < m \le M} \hfill \\ \end{array} } \right.$$
  1. The time from the initial position of the hoist at the end of the tunnel to the target cargo level, i.e. from $m$ level to ${m}^{\mathrm{^{\prime}}}$, is:
    6 $$t_{{mm^{\prime}}} = \left\{ {\begin{array}{*{20}l} {\sqrt {\frac{{4\left| {m - m^{\prime}} \right|H}}{{a_{R} }}} } \hfill & {~\left| {m - m^{\prime}} \right| \le L_{{Em}} } \hfill \\ {\frac{{\left| {m - m^{\prime}} \right|H}}{{V_{{Em}} }} + \frac{{V_{{Em}} }}{{a_{E} }}} \hfill & {L_{{Em}} \le \left| {m - m^{\prime}} \right| \le M} \hfill \\ \end{array} } \right.$$

1. The time-based scheduling model for inbound operations

*Case 1* When there is an idle four-way shuttle on the floor where the target cargo position of the goods to be warehoused is located and the system has an available hoist at the same time, analyse the four-way shuttle and hoist operation steps and calculate the operation time. When the target position is in the warehouse layer, the operation time the hoist takes to carry the goods is 0.

Four-way shuttle operation steps: 1. Shuttle start position is a no-load operation to the end of the aisle elevator mouth. 2. Shuttle car sends goods to the target position.

Hoist operation steps: 1. Hoist runs empty to the warehouse floor. 2. Hoist transports goods to the target cargo floor.

The system works in parallel: the four-way shuttle operation step 1 and the hoist operation steps 1 and 2 execute the operation in parallel at the same time.

The operation time is calculated using Eq. (7).

7 $$\left\{ {\begin{array}{*{20}l} {T_{1} = max\left\{ {\left( {T_{{1Ei}} + T_{{2Ei}} ,T_{{1Rj}} } \right)} \right\} + T_{{2Rj}} } \hfill \\ {1 \le E_{{avaliable}} \le E_{{count}} } \hfill \\ {1 \le R_{{avaliable}} \le R_{{count}} } \hfill \\ {M_{J} = M_{R} } \hfill \\ {T_{{uRj}} = \mathop \sum \limits_{1}^{n} t_{{xx^{\prime}}} + \mathop \sum \limits_{1}^{n} t_{{yy^{\prime}}} ~~\left( {u = 1,2} \right)} \hfill \\ {T_{{wEi}} = t_{{mm^{\prime}}} ~~\left( {w = 1,2} \right)} \hfill \\ \end{array} } \right.$$

If the target location is on the first floor of the warehouse, and ${T}_{1Ei}, {T}_{2Ei}$ are 0.where the four-way shuttle operation step 1 and hoist operation steps 1 and 2 are parallel operations. Before the four-way shuttle completes step 1, it first needs to wait for the hoist operation; before the hoist completes steps 1 and 2, it first needs to wait for the four-way shuttle to complete operation step (1); thus, the operation time that takes the longest will be the completed part.

*Case 2* When there is no free four-way shuttle on the floor where the target position of the goods that need to be stored is located but there is a free four-way shuttle on other floors, and there is an available hoist in the system, analyse the four-way shuttle and hoist operation steps and calculate the operation time.

Four-way shuttle operation steps: 1. The shuttle starts at the starting position and runs empty to the end of the aisle at the hoist entrance. 2. The shuttle follows the hoist to the layer where the target cargo position is located and then carries the cargo to the target position.

Hoist operation steps: 1. The hoist runs empty to the layer where the four-way shuttle is located. 2. The hoist carries the shuttle to the layer where the target cargo is located. 3. The hoist runs empty to the warehouse floor. 4. The hoist carries the cargo to the layer where the target cargo is located.

The system works in parallel: the four-way shuttle operation step 1 and the hoist operation step 1 perform operations in parallel.

The operation time is calculated using Eq. (8).

8 $$\left\{ {\begin{array}{*{20}l} {T_{2} = max\left\{ {T_{{3Ei}} ,T_{{1Rj}} } \right\} + T_{{4Ei}} + T_{{1Ei}} + T_{{2Ei}} + T_{{2Rj}} } \hfill \\ {E_{{avaliable}} = 1} \hfill \\ {1 \le R_{{avaliable}} \le R_{{count}} } \hfill \\ {M_{J} \ne M_{R} } \hfill \\ {T_{{uRj}} = \mathop \sum \limits_{1}^{n} t_{{xx^{\prime}}} + \mathop \sum \limits_{1}^{m} t_{{yy^{\prime}}} ~~\left( {u = 1,2} \right)} \hfill \\ {T_{{wEi}} = t_{{mm^{\prime}}} ~~\left( {w = 1,2,3,4} \right)} \hfill \\ \end{array} } \right.$$

*Case 3* There is no free four-way shuttle in the layer where the target cargo position is located but there are free four-way shuttles in other layers, and there are more than two available hoists in the system analysing the four-way shuttle and hoist operation steps and calculating the operation time.

Four-way shuttle operation steps: 1. Shuttle start position is unloaded and runs to the end of the lane hoist mouth. 2. Shuttle with the hoist to the target cargo level where the layer after carrying goods to the target cargo level.

The hoist operation steps are divided into hoist 1 and hoist 2 operations.

Hoist 1 operation steps: 1. Hoist no-load operation to the four-way shuttle where the layer is. 2. Hoist transports shuttle to the target cargo level where the layer is.

Hoist 2 operation steps: 1. Hoist runs empty to the warehouse floor. 2. Hoist delivery shuttle to goes to the target level.

The system works in parallel: four-way shuttle operation step 1 and hoist 1 operation step 1 execute operations in parallel at the same time. Hoist 1 operation steps 1 and 2 and hoist 2 operation steps 1 and 2 execute operations in parallel at the same time. The operation time is calculated using Eq. (9).

9 $$\left\{ {\begin{array}{*{20}l} {T_{3} = max\left\{ {\max (T_{{3Ei}} ,T_{{1Rj}} + T_{{4Ei}} )} \right\}\left( {T_{{1Ek}} + T_{{2Ek}} } \right) + T_{{2Rj}} ~\left( {i \ne k} \right)} \hfill \\ {E_{{avaliable}} \ge 2} \hfill \\ {M_{J} \ne M_{R} } \hfill \\ {T_{{uRj}} = \mathop \sum \limits_{1}^{n} t_{{xx^{\prime}}} + \mathop \sum \limits_{1}^{m} t_{{yy^{\prime}{\text{~~}}}} \left( {u = 1,2} \right)} \hfill \\ {T_{{wEi}} = t_{{mm^{{{\text{'~~}}}} }} \left( {w = 1,2,3,4} \right)} \hfill \\ \end{array} } \right.$$

Therefore, the total time to complete a batch of warehousing operations from the start of operations for the first shipment to the completion of warehousing operations for the last shipment.

10 $$Min{T}_{c1}={T}_{E}-{T}_{S}$$

See Eq. (10), where ${T}_{S}$ is the start time of the first inbound task and ${T}_{E}$ is the end time of the last inbound task.

(B) The time-based scheduling model for outbound operations

*Case 1* When there is an idle four-way shuttle on the floor where the goods are picked up and there is an available hoist in the system, the operation steps of the four-way shuttle and the hoist are analysed and the operation time is calculated.

Four-way shuttle operation steps: 1. The shuttle starts running unloaded to the target position to pick up the goods; 2. The shuttle carries the goods to the end of the aisle elevator mouth.

Hoist operation steps: 1. The hoist runs empty to the target cargo level; 2. the hoist transports the cargo to the warehouse level.

Parallel and cooperative system operation: four-way shuttle operation steps 1 and 2 and hoist operation step 1.

The operation time is calculated using Eq. (11).

11 $$\left\{ {\begin{array}{*{20}l} {T_{1}^{{\text{'}}} = max\left\{ {T_{{1Ei}}^{{\text{'}}} ,T_{{1Rj}}^{{\text{'}}} + T_{{2Rj}}^{{\text{'}}} } \right\} + T_{{2Ei}}^{{\text{'}}} } \hfill \\ {E_{{avaliable}} = 1} \hfill \\ {M_{J} \ne M_{R} } \hfill \\ {T_{{uRj}}^{{\text{'}}} = \mathop \sum \limits_{{i = 1}}^{n} t_{{xx^{\prime}}} + \mathop \sum \limits_{{i = 1}}^{m} t_{{yy^{\prime}}} ~~\left( {u = 1,2} \right)} \hfill \\ {T_{{wEi}}^{{\text{'}}} = t_{{mm^{\prime}}} ~~\left( {w = 1,2} \right)} \hfill \\ \end{array} } \right.$$

When the four-way shuttle operation steps 1 and 2 and hoist operation step 1 are parallel operations, when the four-way shuttle first completes steps 1 and 2, it needs to wait for the hoist operation; when the hoist first completes step 1, it needs to wait for the four-way shuttle to complete operation steps 1 and 2; thus, the operation time that takes the longest will be completed.

*Case 2* There is no free four-way shuttle on the layer where the goods are picked up, but there are free four-way shuttles on other layers, and there is an available hoist in the system analysing the four-way shuttle and hoist operation steps and calculating the operation time.

Four-way shuttle operation steps: 1. The shuttle starts at the starting position and runs unloaded to the end of the aisle hoist. 2. The shuttle runs unloaded from the end of the aisle hoist to the target position and picks up the goods. 3. The shuttle carries the goods to the end of the aisle hoist.

Hoist operation steps: 1. The hoist runs empty to the level where the idle four-way shuttle is located; 2. The hoist transports the four-way shuttle to the end-of-aisle hoist entrance of the level where the target cargo position is located. 3. The hoist carries the cargo to the first floor of the warehouse.

Parallel and cooperative operation of the system: four-way shuttle operation step 1 and hoist operation step 1 (see Eq. 12).

12 $$\left\{ {\begin{array}{*{20}l} {T_{2}^{{\text{'}}} = max\left\{ {T_{{3Ei}}^{{\text{'}}} ,T_{{3Rj}}^{{\text{'}}} } \right\} + T_{{4Ei}}^{{\text{'}}} + T_{{2Ei}}^{{\text{'}}} + T_{{1Rj}}^{{\text{'}}} + T_{{2Rj}}^{{\text{'}}} } \hfill \\ {E_{{avaliable}} = 1} \hfill \\ {M_{J} \ne M_{R} } \hfill \\ {T_{{uRj}}^{{\text{'}}} = \mathop \sum \limits_{1}^{n} t_{{xx^{\prime}}} + \mathop \sum \limits_{1}^{m} t_{{yy^{\prime}}} ~~\left( {u = 1,2,3} \right)} \hfill \\ {T_{{wEi}}^{{\text{'}}} = t_{{mm^{\prime}}} ~~\left( {w = 1,2,3,4} \right)} \hfill \\ \end{array} } \right.$$

The expression for the time function ${T}_{2}^{\mathrm{^{\prime}}}$ represents a similar meaning to ${T}_{1}^{\mathrm{^{\prime}}}$

Therefore, the total time to complete a batch of outbound operations is from the start of the operations for the first shipment to the completion of the outbound operations for the last shipment.

13 $$Min{T}_{c1}^{\mathrm{^{\prime}}}={T}_{E}^{\mathrm{^{\prime}}}-{T}_{S}^{\mathrm{^{\prime}}}$$

## Proposed algorithm

According to the established mathematical model of multiple four-way shuttle scheduling, the inbound and outbound tasks are decomposed into the problem selecting shuttles to select lifts in different operation stages. Additionally, the objective optimal solution is calculated using an improved genetic algorithm. According to the task combination encoding and decoding characteristics of the genetic algorithm in the selection of crossover and compilation method optimisation, each group of tasks is sorted through the dynamic road network and time window-based improved ${A}^{*}$ algorithm to navigate the horizontal direction path of the four-way shuttle, seeking a safe and conflict-free optimisation path. Thus, the entire path planning of the operations is complete.

### Encoding and decoding

In the model built for the previous inbound and outbound operations, the four-way shuttle is used in batch operations, Since the maximum number of simultaneous shipping operations is limited by the number of four-way shuttles ${R}_{count}$ in the system or by the number of hoists ${E}_{count}$ in the system, the maximum number of parallel operation tasks in the system is ${C}_{batch}=\mathrm{min}({R}_{count},{E}_{count})$. Then the use of scheduling resources according to the operation process in the system can be decomposed into assigning four-way shuttles and assigning cargo lifters, as well as in the inbound operation case 3. To increase the system throughput rate, the operation task will assigns two lifters at the same time: one to deliver shuttles to the target layer and the other to send cargo to the target layer. For other job cases, only one hoist is assigned to complete the job, and the model in this chapter assigns virtual hoists to them. According to this scheduling model for the processing of system jobs $(\mathrm{1,2},\dots ,N)$, it can be abstracted as coding the job sequence and assigning three times system resources: (1) assigning a four-way shuttle; (2) assigning hoist $B$; and (3) assigning hoist $C$. (If the job does not require a second hoist, a virtual lift is assigned, and no job time is consumed at this stage in the objective function calculation). Thus, according to the system task job serial number, hoist serial number, and four-way shuttle serial number to perform real two-dimensional matrix encoding, the generated matrix row number is $P={J}_{count}/{C}_{batch}$ (noninteger division, the last line completes the 0), and the number of columns is $3{C}_{batch}$, that is, ${W}_{{P}^{*}3{C}_{batch}}$.

14 $$\left(\begin{array}{ccc}{a}_{11}& \cdots & {a}_{1{C}_{batch}}\\ \vdots & \ddots & \vdots \\ {a}_{P1}& \cdots & {a}_{P{C}_{batch}}\end{array}\right)\left(\begin{array}{ccc}{b}_{11}& \cdots & {b}_{1{C}_{batch}}\\ \vdots & \ddots & \vdots \\ {b}_{P1}& \cdots & {b}_{P{C}_{batch}}\end{array}\right)\left(\begin{array}{ccc}{c}_{11}& \cdots & {c}_{1{C}_{batch}}\\ \vdots & \ddots & \vdots \\ {c}_{P1}& \cdots & {c}_{P{C}_{batch}}\end{array}\right)$$

See Eq. (13), In the matrix ${W}_{{P}^{*}3{C}_{batch}}={A}_{{P}^{*}{C}_{batch}}*{B}_{{P}^{*}{C}_{batch}}*{C}_{{P}^{*}{C}_{batch}}$, matrix $A$ is the system job matrix, and its members correspond to the job batch number. Matrix $B$ is the hoist assignment matrix, as shown by the model built in this paper. The $B$ and $C$ matrices are for one operation in the same row, and the matrix cannot have the same hoist number, and the elevator code can be reused in different rows. Matrix $B$ is the lift solution, which indicates the number of hoists used in the system for this operation, $C$ is the additional solution of the elevator, when and only when there is no available four-way shuttle in the system where the task is located, and at the same time, the $C$ matrix is involved in the operation when there are more than two available lifts, that is, the system entry model case 3. To prevent too many invalid solutions from being generated, when the target operation layer and the layer where the four-way shuttle subtask ends in the $A$, $B$ matrix coincide, the corresponding rows of the $C$ matrix are in order of the lift numbers that have not yet been used in matrix B. The purpose of this encoding constraint is to provide an extended solution space for the later operators to use. For example, when $({R}_{count}<{E}_{count})$, the maximum concurrency of the system ${C}_{batch}={R}_{count}$, the column number in each row of matrix $A$ indicates the corresponding four-way shuttle vehicle encoding, and the row elements can be expressed as ${X}_{i}=({a}_{i1},{a}_{i2},\dots ,{a}_{i{C}_{batch}};{b}_{i1},{b}_{i2},\dots {b}_{i{C}_{batch}};{c}_{i1,}{c}_{i2},\dots ,{c}_{i{C}_{batch}})$. If the number of tasks is ${a}_{i3}=48$, then the four-way shuttle with the $No. 3$ is used for this task. ${b}_{i3}=2$ indicates that hoist number 2 works together with the 3rd four-way shuttle in batch $i$, that is, the 4-way shuttle $No. 3$ and hoist $No. 2$ work together in this operation to complete the operation of task $No. 48$, and if the target stock of task $No. 48$ is ${J}_{48}=(\mathrm{4,5},7)$ and the coordinates of shuttle $No. 3$ are ${R}_{3}=(\mathrm{2,3},7)$, which means that the target operation level and the four-way shuttle used in this operation are on the same level 7. When coding or generating the target population ${c}_{i3}=0$. When describing the inbound situation three for the previous chapter. ${c}_{i3}=6$ means that the task of this number 48 is completed by the four-way shuttle car $No. 3$, hoist $No. 2$, and hoist $No. 6$ in cooperation The initial position of the shuttle car at this time is located in the layer and the target shelf location of the task is not in the same layer.

In the code for the two-dimensional real matrices created above, each matrix represents an individual, where the three matrices $A,B,C$ represent different chromosomes of the individual, and the different codes are equivalent to various genes. The more common way of generating populations from individuals is the random number production method, and the model constraints listed in this paper are used in order to avoid generating invalid individuals, so this paper uses the random production method with constraints. When $({R}_{count}<{E}_{count})$, matrix $A$ is $(\mathrm{1,2},\dots ,N)$ which is a random combination of different task serial numbers, and each row in matrices $B$ and $C$ is a random combination of lift-and-fall codes but a single row cannot be repeated.

### Adaptation function

In this paper, we adopt the maximisation convention that "the smaller the value of the objective function, the higher the degree of adaptation." The time consumed by the inbound and outbound operations in the system batch is a nonnegative value, which can be set as the objective function's solution target. As seen in the coding model, we know that the system uses batch job $(\mathrm{1,2},\dots ,P)$ batch. Batch sequential occupation of system resources flows the operation. When the resources in Batch 1 complete the job, they can be released and assigned to the tasks represented by the same columns in the following batches for processing. The tasks in different rows of the system may overlap in time. For example, 1. ${a}_{i3}=48$ indicates that $task 48$ has assigned the four-way shuttle $No.3$ in the batch; 2. ${a}_{i3}=96$ indicates that $task 96$ has assigned the four-way shuttle $No.4$ in the batch; 3.${a}_{(i+1)4}=52$ indicates that $task 52$ has assigned four-way shuttle $No.4$ in batch $i+1$. Then the four-way shuttle $No.4$ completes $task 96$ and continues with $task 52$. If the execution of $task 48$ by shuttle 3 takes much longer than $task 96$, $tasks 48$ and $52$ in batches $i$ and $i+1$ to which they belong will overlap in time. In this paper, we choose the difference between the start time ${T}_{1}$ of the total batch (batch $1$) and the end time of the last task in the last batch (batch $P$) as the objective function ${T}_{sum}={T}_{P}-{T}_{1}$, which can be decomposed into the maximum value of the sum of the tasks completed in each column of the matrix of the coding model $A ,$ as shown in Eq. (15).

15 $$\left\{ {\begin{array}{*{20}l} {T_{{sum}} = \max \left( {T_{1} ,T_{2} , \ldots ,T_{{C_{{batch}} }} } \right)} \hfill \\ {T_{j} = \mathop \sum \limits_{{i = 1}}^{P} t_{{ij}} } \hfill \\ \end{array} } \right.$$

The size of the adaptation function is determined to have great relevance to the specific meaning of the problem object to be solved, and in general, the adaptability is obtained by transforming the objective function. The methods of solving the adaptability from the objective function can be divided into direct linear, exponential, and power exponential transformations of the objective function, truncation of the objective function value, etc., to obtain its corresponding adaptability. All these different transformations can be influential and yield efficient population diversity and algorithm convergence^32–35^.

Here, the adaptation function is chosen to be obtained by performing a simple power index transformation from the objective function (see Eq. 16).

16 $$fitness\left(i\right)=f\left(i\right)={(\frac{1}{{T}_{sum}(i)})}^{2}$$

### Selecting arithmetic

The model built and the adaptation function taken in this paper are nonnegative, and the selection operator can be used in the roulette selection method. Roulette selection is essentially a random sampling method with put-back. Individuals of the population are mapped into intervals of the corresponding length according to their adaptability, and the length of each individual's interval is proportional to its adaptability value. The higher the adaptability, the higher the individual’s chance of being selected in the selection operation. A random number is generated, and the corresponding individual is selected according to the interval in which it falls; then, the process is repeated until the desired number of individuals is obtained.

According to adaptability, the selection probability of each individual can be seen in Eq. (17) as follows:

17 $$P({x}_{i})=\frac{fitness({x}_{i})}{\sum_{j=1}^{{N}_{group}}fitness(j)}$$

The cumulative probability of each individual is calculated from the selection probability (see Eq. 17) as follows:

18 $${Q}_{i}=\sum_{i=1}^{{N}_{group}}p({x}_{i})$$

The cumulative probability corresponds to the size of the sector on the turntable, and the larger the sector area, the easier it is to select. Where $P({x}_{i})$ is the selection probability of individual $i$,$fitness({x}_{i})$ denotes the fitness value of individual $i$, and ${N}_{group}$ denotes the total number of individuals in the population.

We select any 6 individuals, and the probability of selection for each individual is The selection probabilities and cumulative probabilities for each individual are shown in Table 1.

**Table 1:** Selection probabilities and cumulative probabilities of individuals.

Individuals	1	2	3	4	5	6
Selection probability	0.095	0.182	0.051	0.308	0.233	0.130
Cumulative probability	0.095	0.277	0.328	0.636	0.87	1

**Figure 3:**
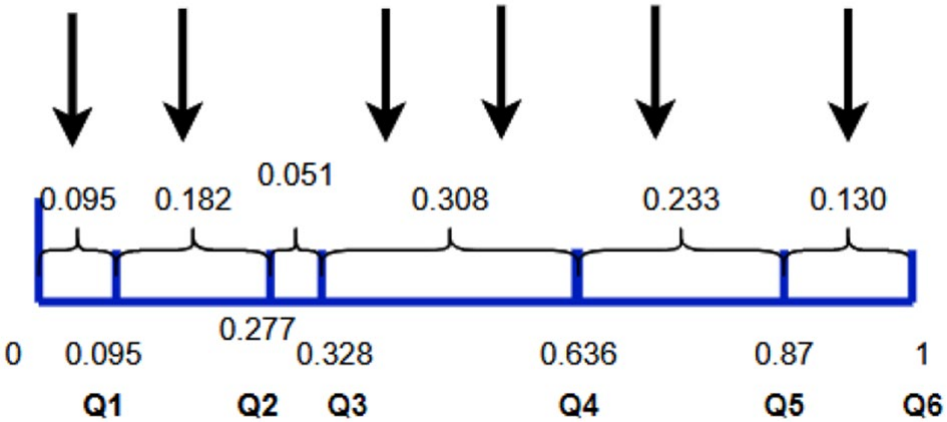
A line segment is used to represent the cumulative probability of the calculated individuals, and the calculated cumulative probability values of the individuals are labeled on a line segment of length 1 by setting a 0 point at the start point of the left end. Six numbers were randomly generated on the interval (0,1), and as seen in the figure, the selected individuals were 1, 2, 4, 4, 5, and 6. Individual number 4 was selected twice.

After calculating the cumulative probability, the calculated individual cumulative probability value is labelled on a line segment of length 1 by setting a 0 point at the start of the left end. In the interval $(\mathrm{0,1})$, 6 numbers are randomly generated, and the selected individuals are $1, 2, 4, 4, 5, 6. No.4$ is selected twice, and the probability of the selected individuals is large, the probability of the selected individuals is large. The small individuals may not be selected, and there is a possibility that all the selected individuals are the same. The randomly generated individual numbers are shown in Fig. 3.

### Crossover operator

In this paper, the individual coding consists of three matrices: for example, when the number of four-way shuttles in the system ${R}_{count}$ is 3, the number of operational tasks ${J}_{count}$ is 9, and the number of hoists in the system ${E}_{count}$ is 4. The individual coding is three sets of $3*3$ matrices $ABC,$ can be seen in Eq. (19).

19 $${A}_{3\times 3}{B}_{3\times 3}{C}_{3\times 3}=\left(\begin{array}{ccc}{a}_{11}& {a}_{12}& {a}_{13}\\ {a}_{21}& {a}_{22}& {a}_{23}\\ {a}_{31}& {a}_{32}& {a}_{33}\end{array}\right)\left(\begin{array}{ccc}{b}_{11}& {b}_{12}& {b}_{13}\\ {b}_{21}& {b}_{22}& {b}_{23}\\ {b}_{31}& {b}_{32}& {b}_{33}\end{array}\right)\left(\begin{array}{ccc}{c}_{11}& {c}_{12}& {c}_{13}\\ {c}_{21}& {c}_{22}& {c}_{23}\\ {c}_{31}& {c}_{32}& {c}_{33}\end{array}\right)$$

The $A$ matrices in the population individuals are task coding matrices, and each $A$ matrix is a sorted combination of task codes. Each row in $BC$ is coded as a lift, and different rows are coded for different job batches. According to this encoding feature, two points of intersection are chosen in this paper, and the intersection points are matrix $A$ and matrix $BC$. In the intersection operation, the matrix is transformed by the row to one-dimension and the two-dimensional matrix, for example (see Eq. 20):

20 $${A}_{3\times 3}=\left(\begin{array}{ccc}{a}_{11}& {a}_{12}& {a}_{13}\\ {a}_{21}& {a}_{22}& {a}_{23}\\ {a}_{31}& {a}_{32}& {a}_{33}\end{array}\right)={A}_{1\times 9}^{\mathrm{^{\prime}}}\left(\begin{array}{ccccccccc}{a}_{12}& {a}_{12}& {a}_{13}& {a}_{21}& {a}_{22}& {a}_{23}& {a}_{31}& {a}_{32}& {a}_{33}\end{array}\right)$$

**Figure 4:**
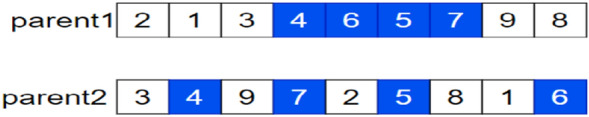
Genetic mapping of the parent at the time of gene crossover operations.

In the crossover operation, by two different parent individuals ${P}_{3\times 9}^{1}={A}_{3\times 3}^{p1}{B}_{3\times 3}^{p1}{C}_{3\times 3}^{p1}$ and ${P}_{3\times 9}^{2}={A}_{3\times 3}^{p2}{B}_{3\times 3}^{p2}{C}_{3\times 3}^{p2}$, respectively, the three sets of matrix values interchangeably crossover to generate new individuals ${N}_{3\times 9}^{1}={A}_{3\times 3}^{n1}{B}_{3\times 3}^{n1}{C}_{3\times 3}^{n1}$, where the two parent matrices mutually operation to generate the child matrix $A$, that is, the parent of the two $A$ matrices crossover reorganisation to generate the child matrix $A$. $BC$ crosses in the parent generation to generate the $BC$ in the child generation, which are two points of crossover in the intersection of the $A$ matrix and $BC$ matrix respectively. To ensure that the crossover is encoded with actual meaning where no crossover operation is performed in between. The two crossovers are performed separately to complete one crossover operation of the individual^36^. The steps are as follows:

Step 1: For the task encoding two-dimensional matrix, ${A}_{3\times 3},$ in the crossover process as seen above the two-dimensional matrix is converted into a single row of one-column real matrix encoding ${A}_{1\times 9}^{\mathrm{^{\prime}}}$ by row expansion, the crossover is completed to produce children, and then its inverse operation is performed to convert from a one-dimensional matrix to a two-dimensional matrix. In the process of the single row one-dimensional matrix operation, subtour exchange crossover is used to avoid the problem of gene duplication in individuals after a simple exchange crossover. The specific operation procedure is shown in Fig. 4 and Eq. (21a and b).

21a $$parent1:{A}_{3\times 3}=\left(\begin{array}{ccc}2& 1& 3\\ 4& 6& 5\\ 7& 9& 8\end{array}\right)={A}_{1\times 9}^{\mathrm{^{\prime}}}\left(\begin{array}{ccccccccc}2& 1& 3& 4& 6& 5& 7& 9& 8\end{array}\right)$$

21b $$parent2:{A}_{3\times 3}^{\mathrm{^{\prime}}}=\left(\begin{array}{ccc}3& 4& 9\\ 7& 2& 5\\ 8& 1& 6\end{array}\right)={A}_{1\times 9}^{\mathrm{^{\prime}}\mathrm{^{\prime}}}\left(\begin{array}{ccccccccc}3& 4& 9& 7& 2& 5& 8& 1& 6\end{array}\right)$$

**Figure 5:**
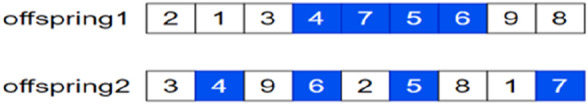
The genetic map of the two offspring generated during the gene crossover operation.

Generate two offspring (see Fig. 5 and Eq. 22a and b):

22a $$offspring1:{A}_{3\times 3}^{o}=\left(\begin{array}{ccc}2& 1& 3\\ 4& 7& 5\\ 6& 9& 8\end{array}\right)$$

22b $$offspring2:{A}_{3\times 3}^{{o}^{\mathrm{^{\prime}}}}=\left(\begin{array}{ccc}3& 4& 9\\ 6& 2& 5\\ 8& 1& 7\end{array}\right)$$

Step 2: For the lifter encoding two-dimensional matrix ${BC}_{3\times 6}$, according to the actual meaning of the encoding, there must not be duplicate encoding in its rows, and the same encoding can be used in various rows (i.e., different task batches). Different parents of the two individuals selected for crossover operations are randomly selected in two rows, and two different individuals are generated by a subtrace interchange crossover (See Eq. 23a and b).

23a $$parent1:{BC}_{3\times 6}=\left(\begin{array}{ccc}4& 3& 2\\ 2& 3& 1\\ 1& 3& 2\end{array}\right)\left(\begin{array}{ccc}0& 1& 0\\ 4& 0& 0\\ 4& 0& 0\end{array}\right)$$

23b $$parent2:{BC}_{3\times 6}^{\mathrm{^{\prime}}}=\left(\begin{array}{ccc}3& 1& 4\\ 4& 2& 1\\ 2& 1& 3\end{array}\right)\left(\begin{array}{ccc}2& 0& 0\\ 0& 3& 0\\ 4& 0& 0\end{array}\right)$$

**Figure 6:**
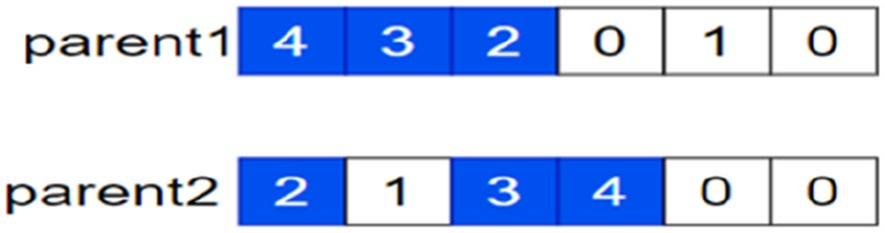
The first row of $parent 1$ and the third row of $parent 2$ were selected for crossover, and the parental gene map at this point.

**Figure 7:**
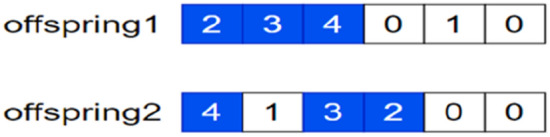
The first row of $parent 1$ and the third row of $parent 2$ are selected for crossover, at which point two offspring gene maps are generated.

Select any row in each of $parent 1$ and $parent 2$ for crossover, and if the first row of $parent1$ and the third row of $parent 2$ are selected, the parent genes are as follows (see Fig. 6):

Generate two offspring (see Fig. 7):

Generate two offspring individuals (see Eq. 24a and b).

24a $$offparent 1:{BC}_{3\times 6}=\left(\begin{array}{ccc}2& 3& 4\\ 2& 3& 1\\ 1& 3& 2\end{array}\right)\left(\begin{array}{ccc}0& 1& 0\\ 4& 0& 0\\ 4& 0& 0\end{array}\right)$$

24b $$offparent 2:{BC}_{3\times 6}^{\mathrm{^{\prime}}}=\left(\begin{array}{ccc}3& 1& 4\\ 4& 2& 1\\ 4& 1& 3\end{array}\right)\left(\begin{array}{ccc}2& 0& 0\\ 0& 3& 0\\ 2& 0& 0\end{array}\right)$$

To avoid the problem of too many invalid solutions in the generated subindividuals, which leads to slow or difficult convergence, the additional solution matrix $C$ of the generated new individuals is also constrained in the crossover process to avoid too many invalid solutions. The solutions that do not satisfy case 3 of the incoming model are discarded and the crossover continues until a satisfying individual is generated.

After performing steps 1 and 2, two new individuals are obtained as follows (see Eq. 25a and b):

25a $${P}_{3\times 9}^{1}={A}_{3\times 3}^{{P}^{1}}{B}_{3\times 3}^{{P}^{1}}{C}_{3\times 3}^{{P}^{1}}=\left(\begin{array}{ccc}2& 1& 3\\ 4& 7& 5\\ 6& 9& 8\end{array}\right)\left(\begin{array}{ccc}2& 3& 4\\ 2& 3& 1\\ 1& 3& 2\end{array}\right)\left(\begin{array}{ccc}0& 1& 0\\ 0& 0& 0\\ 0& 0& 0\end{array}\right)$$

25b $${P}_{3\times 9}^{2}={A}_{3\times 3}^{{P}^{2}}{B}_{3\times 3}^{{P}^{2}}{C}_{3\times 3}^{{P}^{2}}=\left(\begin{array}{ccc}3& 4& 9\\ 6& 2& 5\\ 8& 1& 7\end{array}\right)\left(\begin{array}{ccc}3& 1& 4\\ 4& 2& 1\\ 4& 1& 3\end{array}\right)\left(\begin{array}{ccc}2& 0& 0\\ 0& 3& 0\\ 2& 0& 0\end{array}\right)$$

The child trace swap crossover method is used to select a genome in one parent and find the position of these selected genes in the other parent while keeping the unselected genes unchanged and swapping the positions in the chromosomal genes of the two parents in the order of the selected genes to generate two offspring. This method avoids the problem of task duplication after crossover.

### Variation operator

In this paper, the specific combinations in the coding model have particular meanings and corresponding constraints, such as task number, lift, and ladder coding. For the compilation method, the reciprocal mutation method is used. In the combinatorial optimisation problem, the value of each bit of the gene is unique and called "permutation coding." This means that this feature of the gene must be maintained even after the mutation operation; otherwise, the resulting offspring will be invalid solutions. The reciprocal mutation algorithm randomly identifies two fragments in an individual's gene and performs reciprocal mutation. These two segments must contain the same amount of code. This is shown in the following (see Eq. 26):

26 $${P}_{3\times 9}={A}_{3\times 3}{B}_{3\times 3}{C}_{3\times 3}=\left(\begin{array}{ccc}2& 1& 3\\ 4& 6& 5\\ 7& 9& 8\end{array}\right)\left(\begin{array}{ccc}4& 3& 2\\ 2& 3& 1\\ 1& 3& 2\end{array}\right)\left(\begin{array}{ccc}0& 1& 0\\ 4& 0& 0\\ 4& 0& 0\end{array}\right)$$

We arbitrarily select a row of and matrices; if the second row of the matrix is selected, the first and third columns of the matrix are swapped, and the first and second columns of the matrix are swapped.

The interchanged genetic codes are as follows (see Eq. 27):

27 $${P}_{3\times 9}={A}_{3\times 3}{B}_{3\times 3}{C}_{3\times 3}=\left(\begin{array}{ccc}2& 1& 3\\ 5& 6& 4\\ 7& 9& 8\end{array}\right)\left(\begin{array}{ccc}4& 3& 2\\ 3& 2& 1\\ 1& 3& 2\end{array}\right)\left(\begin{array}{ccc}0& 1& 0\\ 4& 0& 0\\ 4& 0& 0\end{array}\right)$$

### Improvement of the genetic algorithm

For the established time-minimal based multiple four-way shuttle multilift access operation optimisation and path optimisation mathematical model, the basic genetic algorithm, whose probability in crossover and variation operations is fixed, may have difficulty achieving good convergence for input in different operation tasks. Therefore, in this paper, its crossover and variation operators are sampled separately in evolution and adjusted for automatic adaptation. The individual matrix encoding containing information about the job task, the four-way shuttle and the hoist are designed. The genetic operators are designed according to their encoding to improve the operator probability and the fitness function in the algorithm. The mathematical formulae for the crossover operator ${P}_{c}$ and variation operator ${P}_{m}$ in the adaptive genetic algorithm designed in this paper are as follows^37–42^ (see Eq. 28a and b).

28a $$p_{c} = \left\{ {\begin{array}{*{20}l} {k_{1} *\frac{{f_{{max}} - f}}{{f_{{max}} - f_{{agv}} }}~,} \hfill & {f > f_{{agv}} } \hfill \\ {k_{2} ,~} \hfill & {f \le f_{{agv}} } \hfill \\ \end{array} } \right.$$

28b $$p_{m} = \left\{ {\begin{array}{*{20}l} {k_{3} *\frac{{f_{{max}} - f^{\prime}}}{{f_{{max}} - f_{{agv}} }}~,} \hfill & {f^{\prime} > f_{{agv}} } \hfill \\ {k_{4} ,} \hfill & {f^{\prime} \le f_{{agv}} } \hfill \\ \end{array} } \right.$$

It can be seen from the formula that a larger crossover and variation probability is chosen in the beginning stage so that the rough search process is conducive to maintaining population diversity, and later it is adjusted to smaller values for a detailed search to prevent destroying the optimal solution and speed up convergence. The convergence test of the improved algorithm is tested using the test functions listed in Table 2.

**Table 2:** The convergence test of the improved algorithm is tested using the test functions listed in the table.

Function name	Formula	Minimum value	Definition domain
Ackley	$f\left(x,y\right)=-20\mathit{exp}\left(-0.2\sqrt{0.5\left({x}^{2}+{y}^{2}\right)}\right)-\mathit{exp}\left(0.5\left(\mathrm{cos}\left(2\pi z\right)+\mathrm{cos}\left(2\pi y\right)\right)\right)+20+e$	0	$(-5\le x,y\le 5)$
Sphere	$f\left(x\right)=\sum_{i}^{n}{x}_{i}^{2}$	0	$x\subset (-\infty ,+\infty )$ $i\subset (1,n)$
Rosenbrock	$f\left(x\right)=\sum_{i=1}^{n-1}\left[100{({x}_{i+1}-{x}_{i}^{2})}^{2}+{({x}_{i}-1)}^{2}\right]$	0	$x\subset (-\infty ,+\infty )$ $i\subset (1,n)$

**Figure 8:**
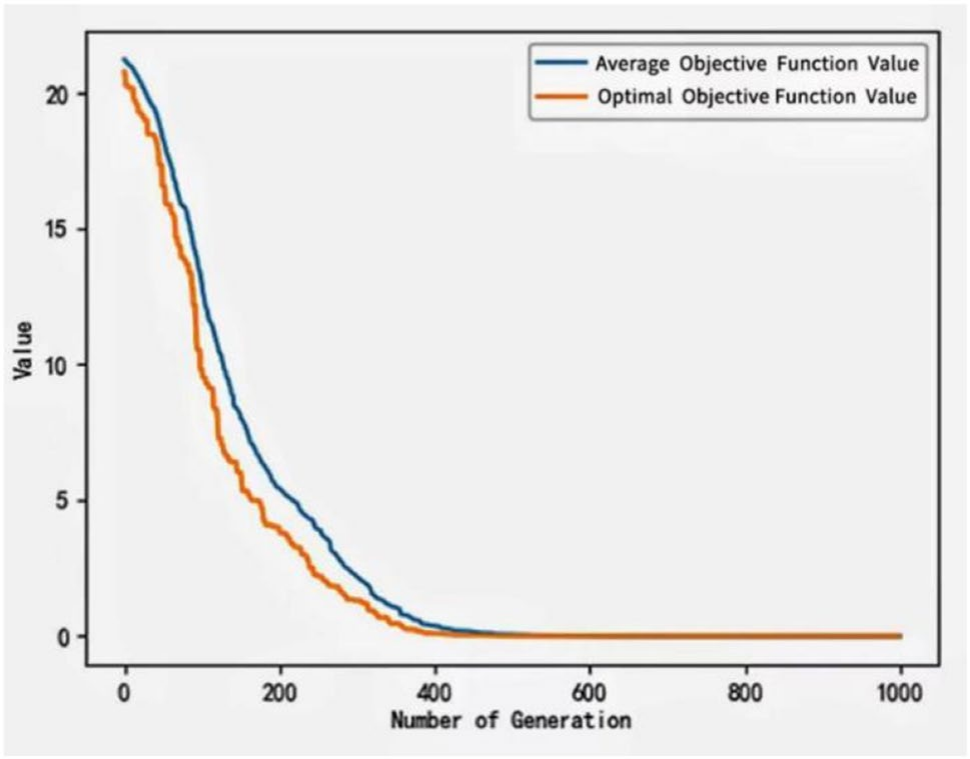
Convergence curve of the improved algorithm tested with Ackley's function.

**Figure 9:**
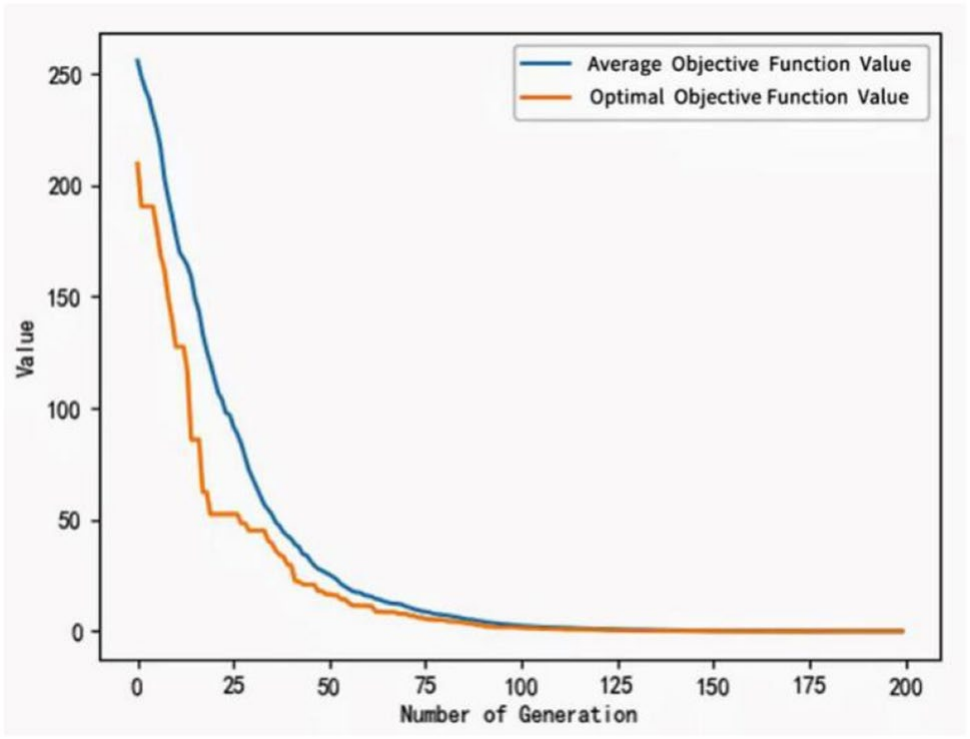
Test the function convergence curve of the improved algorithm with the Sphere test function.

**Figure 10:**
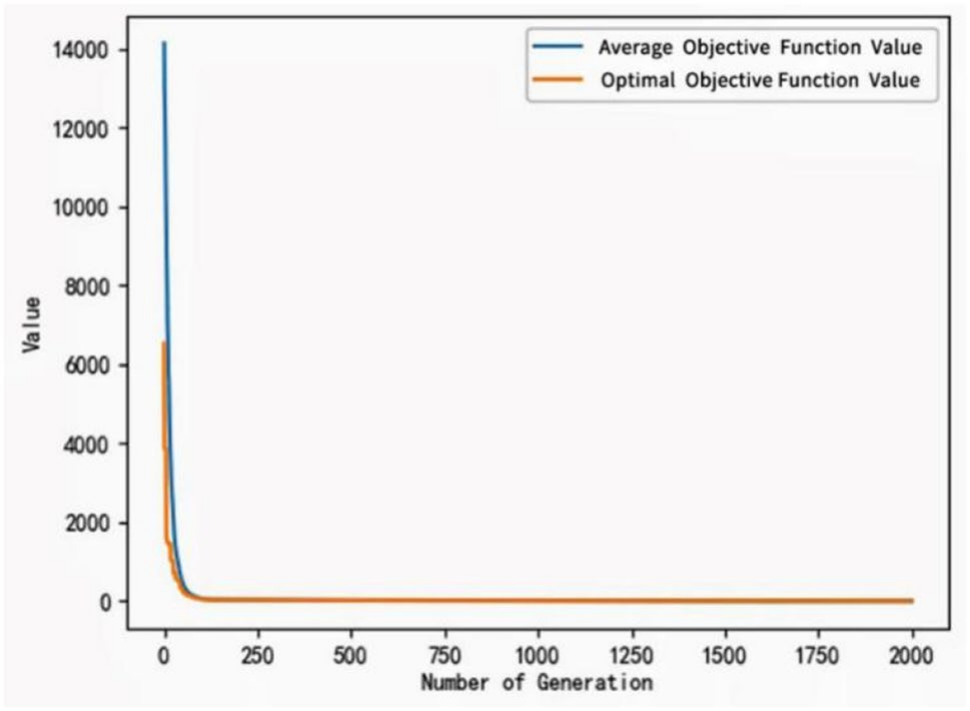
Test the function convergence curve of the improved algorithm with Rosenbrock test function.

The test function convergence curve is shown in Figs. 8, 9, and 10.

### Application of the A* algorithm to shuttle navigation

The conflicts that often occur in four-way shuttles are node conflicts, phase conflicts, catch-up conflicts, and blocking conflicts. In the model established by the system, the four-way shuttles of the same layer work together dynamically at the same time, and the physical characteristics of the warehouse in the same layer are aisles and shelves composed of a matrix mesh structure. The shuttle track can be naturally abstracted into a diagram when scheduling the four-way shuttles of the same layer, and each turning point corresponds to a vertex in the diagram. Each travelable distance from acceleration to a standstill in every section before turning can be abstracted into a diagram of an arc. Solving the inbound task of the shuttle from the lift entrance to the cargo path or the outbound task of the cargo path to the lift entrance can be transformed into the problem of solving the shortest path in a directed acyclic weighted graph. Based on the comprehensive shuttle physical structure of the characteristics of the warehouse, this paper selects the heuristic ${A}^{*}$ algorithm^43–46^, using the Euclidean distance as the valuation function calculation, which can avoid dropping invalid points and accelerate the finding efficiency. Because the warehouse construction is completed and its four-way shuttle physical track is fixed, the path finding task calculation of the four-way shuttle is accelerated by statically initialising the graph of the road network in each layer when the system is started, and only the weights of the arcs that can be directly reached from the starting position of the four-way shuttle need to be amended. The problem of shuttle collisions can be cleverly avoided by performing a time window correction of the arc weights in the graph using the time that the four-way shuttle occupies the track in the process of the scripture-seeking calculation. In this paper, the four-way shuttle path is globally scheduled by the backend system, the four-way shuttle uses wireless devices to communicate with the backend, and the four-way shuttle is equipped with a distance-measuring radar collision avoidance device. For tasks in the same batch, the four-way shuttle's code number is used as the order of calculating the path, and the smaller the code the higher the priority.

**Figure 11:**
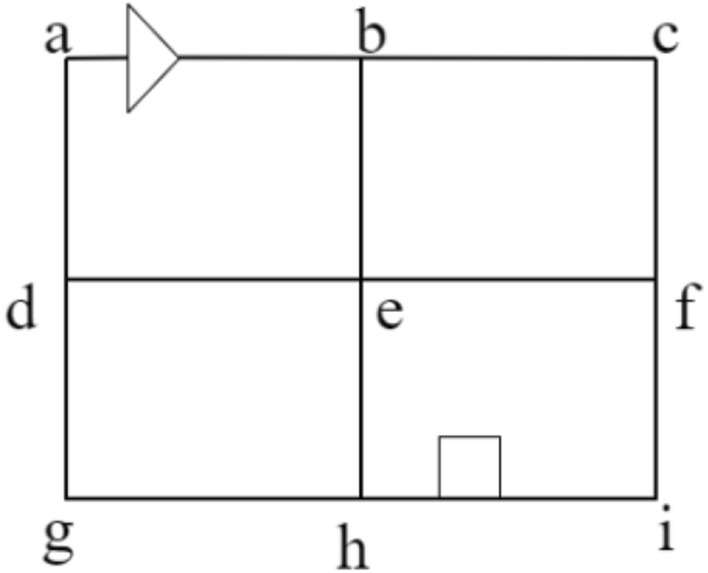
Four-way shuttle access path weighting diagram (the triangle in the diagram represents the four-way shuttle location, the square represents the access cargo position).

**Figure 12:**
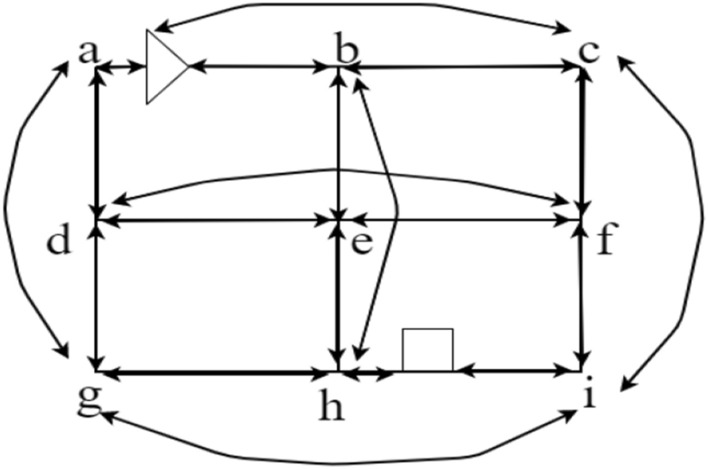
The path weights that the four-way shuttle can travel to access the goods can be used to find the minimum path containing a set of arcs and vertices in this weight map by the A* algorithm.

In the current task to be performed operations, we let the initial position of the four-way shuttle ((${x}_{i},{y}_{j},{z}_{k}$) (assuming that the four-way shuttle position between nodes $a,b$ point for $w$), to reach the destination position for (${x}_{i}^{\mathrm{^{\prime}}},{y}_{i}^{\mathrm{^{\prime}}},{z}_{k}^{\mathrm{^{\prime}}}$) (assuming that the target position between points $h,i$ point for $q$). Its weight map can be amended according to the location of the four-way shuttle and can be direct to the point, adding the starting position of the four-way shuttle and the target position as a vertex, Additionally, the arc that these two vertices can reach directly without turning is amended (see Figs. 11 and 12).

At this point, the data structure needed to find the minimum path for the first time is completed, and the ${A}^{*}$ algorithm can be used to find the minimum path containing a set of arcs and vertices in the weighted graph above. Based on the established search weight map, it is then possible to perform an iterative search for the minimum distance path according to the ${A}^{*}$ algorithm described above.

$H(n)$ is designed as the Euclidean weighting of the amount of time consumed from point to the target point as follows (see Eq. 28a and b):

29 $$H\left(n\right)=\sqrt{{t}_{x{x}^{\mathrm{^{\prime}}}}^{2}+{t}_{y{y}^{\mathrm{^{\prime}}}}^{2}}$$

### Improved ${{\varvec{A}}}^{\mathbf{*}}$ algorithm based on time windows

The conventional ${A}^{*}$ algorithm may have more than one four-way shuttle in the same layer in the parallel operation of the system, and the path planning of each single unit may lead to collision in the same lane at the same time. In this paper, the graph used for the solution of ${A}^{*}$ is weighted again on the time window, and the constrained paths are solved or planned according to the order of the shuttle vehicle’s execution tasks.

**Figure 13:**
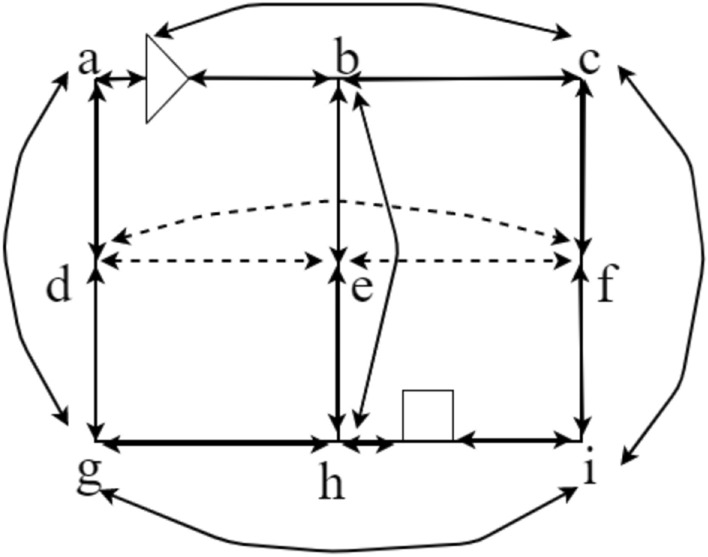
Arcs affected by $$\overset{\lower0.5em\hbox{$\smash{\scriptscriptstyle\frown}$}}{d} e$$ at point e path conflict.

The time window correction rules are as follows: 1. Timing rules based on the time window, monotonically increasing calculation; 2. The time window is calculated as the smallest arc, and the period when the four-way shuttle enters the arc and makes out the arc is the time window of its arc; 3. If its arc  is the trajectory occupied by the four-way shuttle in time $$\left[{T}_{A},{T}_{B}\right]$$, then within this time window, its two nodes, $$A,B$$ risk conflict collision, and the time window updates the global graph of system $$G.$$ The arcs in which $$A,B$$ are located and the arcs that can be reached directly with the help of $$A,B$$ are updated in the time window. If the $$\overset{\lower0.5em\hbox{$\smash{\scriptscriptstyle\frown}$}}{d} e$$ arc in Fig. 13 has a conflict at point e, the weights of the $$d,e$$ arcs and the arcs adjacent to their vertices are increased, and the time consumed by the dashed paths is required to increase the weight, $${ T}_{max}$$.

## Application example analysis

### Data selection

In this paper, by using a four-way shuttle system of an actual warehouse as a reference, we implement the simulation arithmetic examples based on the time-minimised improved genetic algorithm for task operations and the time-window improved A* algorithm for seeking path planning, and analyse the simulation experimental results to draw conclusions on scheduling optimisation. Company *A* is a large enterprise, and one of its warehouses is a $10*20*8$(10 aisles, 20 columns, 8 floors) four-way shuttle storage system. To verify the validity of the model, a batch of in/out operation data is taken, and the parameters of the four-way shuttle storage system are set as shown in Table 3.

**Table 3:** In order to verify the accuracy of the model established in this paper, take the data of a batch of inbound and outbound operation documents of company A as an example, and set the parameters of the four-way shuttle storage system.

Parameters/symbols	Value
Height of each shelf/$H$	1.5 m
Width of individual aisles/${W}_{T}$	1 m
Number of shelves/$M$	8
Length of individual compartments/$L$	1 m
Width of individual compartments/${W}_{C}$	1 m
Number of cargo columns	20
Maximum speed of the four-way shuttle/${V}_{Rm}$	2 m/s
Acceleration of the four-way shuttle/${a}_{R}$	1 m/s^2^
Maximum speed of the hoist/${V}_{Em}$	2 m/s
Acceleration of the hoist/${a}_{E}$	1 m/s^2^

The four-way shuttle system has four four-way shuttles and four hoists in operation. The initial positions of the four-way shuttles are $(\mathrm{4,2},7), (\mathrm{1,7},5), (\mathrm{4,6},5), (\mathrm{3,2},1), (\mathrm{6,5},2)$, respectively for the serial numbers (${R}_{1}$)(${R}_{2}$)(${R}_{3}$)(${R}_{4}$). The locations of the four-way shuttles and hoists are $(\mathrm{2,0},4), (\mathrm{4,0},1), (\mathrm{5,0},3), (\mathrm{6,0},2)$, and their serial numbers are ${E}_{1},{E}_{2},{E}_{3},{E}_{4}$. There is a batch of inbound and outbound tasks in a certain document, which include 2 outbound tasks and 2 inbound tasks, and the specific information of the inbound and outbound tasks is shown in Table 4. We seek a four-way shuttle and hoist scheduling solution that minimises the overall operating path time to complete the batch of tasks.

**Table 4:** List of outgoing/incoming tasks.

Serial number	Alleyway	Number of columns	Number of layers	Outbound/inbound
1	2	7	3	Inbound
2	3	6	4	Outbound
3	4	9	7	Inbound
4	5	11	5	Outbound

### Simulation experiments and data analysis

After the program is run, the iterative process and related data of the two algorithms are obtained, the population size is 20, the maximum number of iterations is 100, the crossover probability of other parameters is 0.5 and the variation probability is 0.004 in the classical genetic algorithm, the self-applicable crossover interval is $[0.5, 0.9]$ and the variation probability interval is $[0.001, 0.0008]$ in the improved genetic algorithm. The two genetic algorithms run 15 times independently.

As shown in Table 5, the improved algorithm has obvious advantages in solution stability and convergence speed, and the convergence efficiency is better than the classical algorithm by obtaining the average value of the optimal solution for the first time. In addition, better stability can be illustrated by the variance and skewness.

**Table 5:** Comparison of convergence between classical algorithm optimal first generation and improved algorithm optimal first generation.

Serial number	Classical algorithm optimal first generation	Improved algorithm optimal first generation
1	80	49
2	77	31
3	61	44
4	47	48
5	84	62
6	53	35
7	45	52
8	56	24
9	57	31
10	72	54
11	69	23
12	65	54
13	39	20
14	83	48
15	26	31
Average	60.9	40.4
Variance	273.1	163.7
Skewness	− 0.36	− 0.08

**Figure 14:**
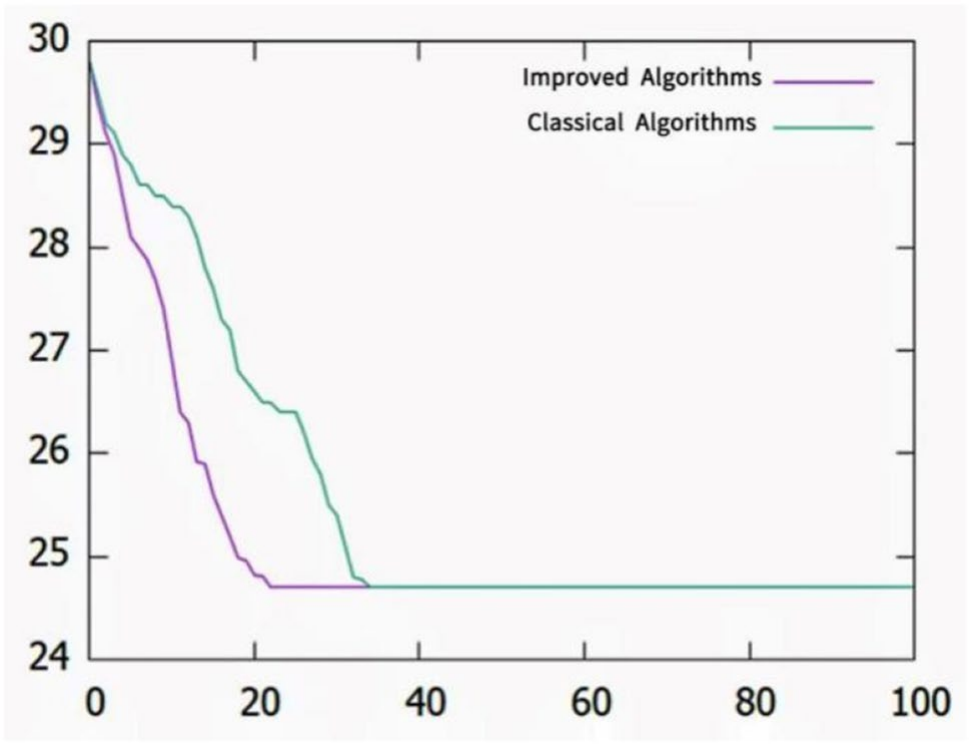
The convergence trend of the improved algorithm and the traditional algorithm shows that the improved algorithm has obvious advantages in solution stability and convergence speed.

As shown in Fig. 14, according to the data input in Table 5, both sets of algorithms can eventually converge to the optimal solution because the maximum number of solution spaces of the model population in this paper is the number of arrangement results of the four-way shuttle and the lift and its tasks, and the individual solutions in each population are discrete. However, the graph shows that in this calculation, the improved algorithm converges to the optimal solution approximately 20 times; however, the classical algorithm becomes optimal approximately 35 times. however, the classical algorithm gets optimal at about 35 times. Only four jobs were used in this simulation task, and the convergence efficiency of the improved algorithm will have more obvious advantages as the scale of the number of tasks increases. According to the algorithm operation results, the optimal solution is obtained as shown in Table 6.

**Table 6:** According to the results of the algorithm, the optimal solution of the job is obtained.

Four-way shuttle serial number	Inbound and outbound task coordinates	Elevator serial number
(${R}_{1}$)	(4,9,7)	${E}_{2}$
(${R}_{2}$)	(5,11,5)	${E}_{1}$
(${R}_{3}$)	(2,7,3)	${E}_{3}$
(${R}_{4}$)	(3,6,4)	${E}_{4}$

In the above table, we can see that the four-way shuttle and hoist scheduling sequence are:$({R}_{1})\to (\mathrm{4,9},7)\to {E}_{1}, ({R}_{2})\to (\mathrm{5,11,5})\to {E}_{2},({R}_{3})\to (\mathrm{2,7},3)\to {E}_{3} , ({R}_{4} )\to (\mathrm{3,6},4)\to {E}_{4}$, where $({R}_{1})$ and $({R}_{3})$ four-way shuttle complete the inbound operation, $({R}_{2})$ and $\left({R}_{4}\right)$ four-way shuttle complete the outbound operation. operations. The total time for the inbound operation is 21.3 s, and the total path is 23 m. The total time for the outbound operation is 24.71 s; the total path is 30 m.

## Conclusion

The main work of this paper is to optimise the scheduling and automatic path finding for the access and egress tasks of the four-way shuttle system. For the four-way shuttle system with multiple four-way shuttles and multiple lifts, we analyse the flow of the four-way shuttle system and construct a mathematical model for scheduling optimisation based on the shortest amount of time. For the model in this paper, the optimised mathematical model is solved using an improved genetic algorithm to solve the task planning and an improved ${A}^{*}$ algorithm to optimise the path within the shelf layer, in combination with the characteristics of the model. Conflict-free optimal paths are sought for four-way shuttles operating in parallel on each layer, and conflict control of the four-way shuttle system is performed using dynamic graph theory and the time-window-based ${A}^{*}$ algorithm. First, this paper establishes an optimisation model based on an improved genetic algorithm for scheduling the shortest time inbound and outbound operation tasks for the storage layout and structural characteristics of the four-way shuttle system. Second, this paper proposes an innovative algorithm design for the parallel operation of a four-way shuttle and hoist and automatic path finding of the four-way shuttle without movement direction limitations. According to the task scheduling optimisation model for inbound and outbound operations, an improved genetic algorithm solution model is proposed. The dynamic road network and the improved algorithm based on time windows are used to avoid obstacles during the operation of multiple four-way shuttles because of the possible overlap of movement paths when multiple four-way shuttles operate in parallel in a single layer and the possible conflict of reaching the overlapping area within the same period.

## Data Availability

Te datasets used and/or analyzed during the current study available from the corresponding author on reasonable request.

## Abbreviations

AS/RS: Automated storage/retrieval system
SBS/RS: Shuttle-based storage and retrieval
FCFS: First-come, first-served
AVS/RS: Automated vehicle storage and retrieval system
R/S: Storage/retrieval
